# Prevalence of HIV-1 drug resistance amongst newly diagnosed HIV-infected infants age 4–8 weeks, enrolled in three nationally representative PMTCT effectiveness surveys, South Africa: 2010, 2011–12 and 2012–13

**DOI:** 10.1186/s12879-019-4339-y

**Published:** 2019-09-16

**Authors:** Gillian M. Hunt, Johanna Ledwaba, Anna Salimo, Monalisa Kalimashe, Thu-Ha Dinh, Debra Jackson, Gayle Sherman, Adrian Puren, Nobubelo K. Ngandu, Carl Lombard, Lynn Morris, Ameena Goga

**Affiliations:** 1 Centre for HIV and STIs, National Institute for Communicable Diseases, National Health Laboratory Services, Johannesburg, South Africa; 2 Faculty of Health Sciences, University of the Witwatersrand, Johannesburg, South Africa; 3 US Centers for Disease Control and Prevention, Center for Global Health, Division of Global HIV/TB, Atlanta, GA USA; 4 School of Public Health, University of the Western Cape, Bellville, South Africa; 5 UNICEF, New York, NY USA; 6 Health Systems Research Unit, South African Medical Research Council, Cape Town, South Africa; 7 Biostatistics Unit, South African Medical Research Council, Cape Town, South Africa; 8 School of Public Health and Family Medicine, University of Cape Town, Cape Town, South Africa; 9 Department of Paediatrics, University of Pretoria, Pretoria, South Africa

**Keywords:** HIV drug resistance genotyping, Dried blood spots, Children

## Abstract

**Background:**

South Africa (SA) has expanded efforts to reduce mother-to-child transmission of HIV (MTCT) to less than 2% at six weeks after birth and to less than 5% at 18 months postpartum by 2016. Despite improved antiretroviral regimens and coverage between 2001 and 2016, there is little data on infant HIV drug resistance. This paper tracks the prevalence of HIV drug resistance patterns amongst HIV infected infants from three nationally representative studies that assessed the effectiveness of national programs to prevent MTCT (PMTCT). The first study was conducted in 2010 (under the dual therapy PMTCT policy), the second from 2011 to 12 (PMTCT Option A policy) and the third from 2012 to 13 (PMTCT Option A policy). From 2010 to 2013, infant non-nucleoside reverse transcriptase inhibitor (NNRTI) exposure increased from single dose to daily throughout breastfeeding; maternal nucleoside reverse transcriptase inhibitor (NRTI) and NNRTI exposure increased with initiation of NNRTI-and NRTI- containing triple antiretroviral therapy (ART) earlier in gestation and at higher CD4 cell counts.

**Methods:**

Three nationally representative surveys were conducted in 2010, 2011–12 and 2012–13. During the surveys, mothers with known, unknown, or no exposure to antiretrovirals for PMTCT and their infants were included, and MTCT was measured. For this paper, infant dried blood spots (iDBS) from HIV PCR positive infants aged 4–8 weeks, with consent for additional iDBS testing, were analysed for HIV drug resistance at the National Institute of Communicable Diseases (NICD), SA, using an in-house assay validated by the Centers for Disease Control and Prevention (CDC). Total viral nucleic acid was extracted from 2 spots and amplified by nested PCR to generate a ~ 1 kb amplicon that was sequenced using Sanger sequencing technologies. Sequence assembly and editing was performed using RECall v3.

**Results:**

Overall, HIV-1 drug resistance was detected in 51% (95% Confidence interval (CI) [45–58%]) of HIV PCR positive infants, 37% (95% CI [28–47%]) in 2010, 64% (95% CI [53–74%]) in 2011 and 63% (95% CI [47–77%]) in 2012 (*p* < 0.0001), particularly to the NNRTI drug class. Pooled analyses across all three surveys demonstrated that infants whose mothers received ART showed the highest prevalence of resistance (74%); 26% (21/82) of HIV PCR positive infants with no or undocumented antiretroviral drug (ARV) exposure harboured NNRTI resistance.

**Conclusions:**

These data demonstrate increasing NNRTI resistance amongst newly-diagnosed infants in a high HIV prevalence setting where maternal ART coverage increased across the years, starting earlier in gestation and at higher CD4 cell counts. This is worrying as lifelong maternal ART coverage for HIV positive pregnant and lactating women is increasing. Also of concern is that resistant virus was detected in HIV positive infants whose mothers were not exposed to ARVs, raising questions about circulating resistant virus. Numbers in this group were too small to assess trends over the three years.

## Introduction

Preventing mother-to-child transmission (PMTCT) of HIV has been a global priority for health research and programmatic implementation over the past two decades. The global scale-up of antiretroviral (ARV) prophylaxis for PMTCT has led to two thirds of HIV-infected pregnant women in resource limited settings receiving ARVs for PMTCT, and resulting in a 48% decline in mother to child HIV transmission between 2009 and 2015 [1]. By 2008, the South African national PMTCT policy recommended maternal combination (triple) antiretroviral treatment (ART), using stavudine (d4T) with lamivudine (3TC) and efavirenz (EFV) or nevirapine (NVP) if CD4 cell count was < 200 cells/mm^3^, or maternal zidovudine (AZT) prophylaxis from 28 weeks gestation with single dose nevirapine (sdNVP) in labour with infant sdNVP and 7 or 28 days of infant AZT. From April 2010, Option A was introduced, wherein lifelong maternal ART, using tenofovir (TDF) with 3TC or emtricitabine (FTC) and NVP (or AZT with 3TC and NVP) was initiated if CD4 cell count was < 350 cells/mm^3^, or AZT prophylaxis offered from 14 weeks gestation with sdNVP and 3 hourly AZT during labour and single dose TDF/FTC postpartum; infants received daily NVP for six weeks (if not breastfed) or till one week post breastfeeding cessation. Under Option A, all HIV-exposed infants received daily nevirapine (NVP) for six weeks if not breastfeeding or until one week following cessation of breastfeeding. This study reports data collected during the implementation of Option A in South Africa. In April 2013, national PMTCT policy transitioned to WHO PMTCT Option B, and in 2015 to PMTCT Option B+; the latter recommends initiation and lifelong continuation of ART amongst all pregnant and lactating women, regardless of their CD4 cell count. Since 2010, antiretroviral treatment guidelines recommended that HIV-infected infants and children less than 3 years of age should be initiated on a protease-inhibitor (PI) based regimen immediately after diagnosis [2, 3].

During the time of this study the South African National Strategic Plan on HIV, TB and STI’s (2012–2016) aimed to reduce the risk of MTCT of HIV to less than 2% at six weeks after birth and to less than 5% at 18 months postpartum by 2016 [4]. To this end, the South African National Department of Health expanded and improved PMTCT services within the maternal and child health program. To assess the effectiveness of the national PMTCT program, three annual, nationally representative, cross-sectional surveys, each called the South African PMTCT Evaluation (SAPMTCTE), were conducted between 2010 and 2013. Each survey aimed to capture HIV-exposed and HIV-unexposed infants at 4–8 weeks of age, regardless of PMTCT exposure. Dried blood spots were collected and tested for HIV antibodies and qualitative polymerase chain reaction (PCR) to determine infant HIV exposure and infection status, respectively. Data demonstrated that rates of early mother to child transmission of HIV declined from 3.5% (2010) to 2.7% (2011–12) and to 2.6% (2012–13) at 6 weeks post-delivery [5–8]. Over the 3 years of surveillance, infant HIV exposure is estimated to have remained constant; approximately 29–32% of mothers reported being HIV positive during pregnancy, and 33% of participating infants were shown to be HIV-exposed at six weeks post-delivery. In addition, access to HIV testing and care was high, with more than 90% of mothers receiving PMTCT interventions.

Resistance to non-nucleoside reverse transcriptase inhibitors (NNRTI) frequently occurs in HIV-infected infants following exposure to sdNVP for PMTCT, increasing the risk of virological failure when HIV PCR positive infants are initiated on NNRTI-containing ART regimens [9]. Early studies on women and infants receiving Option A show lower rates of resistance, yet report increased selection of nucleoside reverse transcriptase inhibitor (NRTI) resistance in women receiving AZT from early (< 20 weeks gestation) in pregnancy [10]. Furthermore, the use of ultra-sensitive assays that detect resistance-associated mutations at low frequencies within the circulating viral subspecies population show even higher proportions of infants harbouring resistance than detected using routine Sanger (not ultra-sensitive) sequencing assays [11, 12]. Drug resistance patterns and frequencies in real-life programmatic settings are less well documented. The objective of this sub-study was to track the prevalence of HIV-1 drug resistance (HIVDR) in nationally representative samples of young infants newly diagnosed with HIV, enrolled in the three SAPMTCTE surveys, and conducted in real-life, programmatic settings.

## Methods

Data for this resistance sub-study were drawn from the three nationally representative, cross-sectional SAPMTCTE, conducted amongst infants aged 4–8 weeks receiving their first immunisation. Data were collected at 580 public primary health care clinics and community health centres in all nine South African provinces between June – December 2010 (2010 survey), August 2011 – March 2012 (2011 survey), and October 2012 – May 2013 (2012 survey), respectively [13]. Caregiver/infant pairs of known or unknown HIV and PMTCT status were consecutively or systematically selected depending on facility size. Data were gathered from patient-held charts (“Road to Health Charts/Booklets”) and during interviews (self-reported maternal HIV testing and HIV status, infant feeding practices and ARV regimen). Infant dried blood spots (iDBS) were collected by heel-prick from all consented infants and assessed for HIV-exposure using a biomedical marker HIV Enzyme immunoassay (EIA). The iDBS with positive EIA or discordant EIA results were compared with self-reported results or were tested using a qualitative total nucleic acid (TNA) PCR to determine infant’s HIV infection. All DBS cards were stored at -20 °C before analysis.

HIVDR genotyping was performed on all adequate HIV PCR positive iDBS specimens from participants that had consented for further testing. Specimens from participants from whom consent for further testing was not received, or iDBS cards with insufficient or unusable blood spots were excluded. Genotyping for resistance mutations to the NRTI, NNRTI and protease inhibitor (PI) drug classes was performed using a validated in-house sequencing method recommended by the CDC [14] as previously described [15]. Briefly, total viral nucleic acid was extracted from two spots and amplified by nested PCR to generate a ~ 1 kb amplicon that was sequenced using Sanger sequencing technologies. Sequence assembly and editing was performed using RECall v3 [16]. Resistance was defined as the presence of mutations associated with impaired drug susceptibility using the Stanford algorithm (https://hivdb.stanford.edu/) and the 2015 IAS-USA drug resistance mutation list [17].

Group comparisons for categorical data were performed using chi-square tests. Binomial regression models were applied to estimate absolute differences in resistance prevalence over the different years and exposure categories. The analyses performed are unweighted and did not take the survey design into account due to the small number of HIV positive participants involved. Summary statistics were calculated using STATA v14 (StataCorp. 2015 Stata Statistical Software: Release 14. College Station, TX: StataCorp LP).

For the purpose of this analysis we categorised maternal and infant antiretroviral access into five main groups: maternal ART with infant prophylaxis (NVP or AZT); maternal ARV prophylaxis with infant prophylaxis (NVP or AZT); infant prophylaxis only (mother reported not receiving any antiretroviral drug), any other antiretroviral combination including maternal ART only or maternal ARV only (no infant prophylaxis) or maternal ART and ARV (not one or the other) for some duration of pregnancy, and no known maternal antiretroviral exposure.

The SAPMTCTE was approved by the institutional review board of the SA Medical Research Council (MRC), study number EC09–002. The project was reviewed according to the CDC human research procedures and was determined to be research, but CDC was not engaged. The resistance sub-analysis study was approved by the Institutional Review Board of the University of the Witwatersrand (M110737).

## Results

Two hundred and eighty-five TNA positive iDBS specimens gathered at 4–8 weeks were identified in the three SAPMTCTE: 125 in the 2010 survey, 89 in 2011, and 71 in 2012. Of these, 116, 82 and 71 infants were eligible for inclusion in this sub-study, respectively. Drug resistance testing was therefore performed in a total of 269 iDBS specimens collected from all three surveys, representing 94% of all positive specimens identified in the surveys. Two specimens were subsequently removed from the 2010 survey set because of unresolved phylogenetic clustering. Of the 267 iDBS tested, PCR and genotyping was successful in 220 (82%) specimens: in 101 (101/114; 89%) specimens from the 2010 survey, 78 (78/82, 95%) from the 2011 survey and 41 (41/71, 58%) specimens from the 2012 survey (Table 1). Forty- seven specimens did not amplify.

**Table 1:** Antiretroviral exposure status of 220 HIV-positive infants analysed for resistance associated mutations over the 3 South African Prevention of Mother to Child Transmission Evaluations

	2010 survey	2011–12 survey	2012–13 survey	Total
Maternal ART plus infant NVP +/− AZT	10 (10%)	11 (14%)	13 (32%)	34 (15%)
Maternal ARV plus infant NVP+/− AZT	33 (33%)	23 (29%)	7 (17%)	63 (29%)
Infant NVP+/−AZT only	11 (11%)	10 (13%)	5 (12%)	26 (12%)
Any other ARV combination	10 (10%)	3 (4%)	2 (5%)	15 (7%)
No/unknown exposure	37 (37%)	31 (40%)	14 (34%)	82 (37%)
Total	101	78	41	220

*ART* triple drug antiretroviral therapy, *ARV*: antiretroviral therapy administered to prevent mother to child transmission

Amongst 220 iDBS sequences analysed, most of the infants (166/220, 75%) were 6 weeks of age at time of specimen collection, whilst 17 (8%) were 4–5 weeks of age, and 37 (17%) were 7–8 weeks of age. One hundred and fifteen (52%) were female, and 56% were ever breastfed. Over three surveys, 34 (15%) infants were exposed to maternal ART and received daily infant NVP ± AZT postpartum, whereas 29% were exposed to maternal ARV for PMTCT with daily infant NVP ± AZT (Table 2). The proportion of HIV PCR positive infants receiving NVP and exposed to maternal ART increased from 10% in 2010 to 32% in 2012, whereas the proportion of infants exposed to maternal ARV prophylaxis decreased from 33 to 17% in the same period. Within this subset, 37% of infants were never exposed to any ARVs or had unknown exposure (Table 2). Unknown exposure was defined as infants whose caregiver declined to answer or did not recall, or data was not recorded on the questionnaire.

**Table 2:** Prevalence of resistance associated mutations to non-nucleoside reverse transcriptase inhibitors (NNRTI) and nucleoside reverse transcriptase inhibitors (NRTI) in 220 HIV-infected infants identified over 3 survey years categorised according to infant PMTCT exposure

Infant PMTCT exposure:	n/N (%) with NNRTI mutations	n/N (%) with NRTI mutations
Maternal ART plus infant NVP ± AZT	25/34 (74%)	5/34 (15%)
Maternal ARV plus infant NVP ± AZT	43/63 (68%)	1/63 (2%)
Infant NVP ± AZT only	17/26 (65%)	1/26 (4%)
Other maternal and/or infant ARV	6/15 (40%)	1/15 (7%)
No/unknown maternal and/or infant ARV exposure	21/82 (26%)	2/82 (2%)
Total	112/220 (51%)	10/220 (5%)

*ART* combination triple drug antiretroviral therapy, *ARV* antiretroviral therapy administered to prevent mother to child transmission

Drug resistance mutations to the NNRTI class of drugs were detected in 51% of 220 specimens; 37% (95% CI [28–46%]) in 2010, 64% (95% CI [53–74%]) in 2011 and 63% (95% CI [48–77%]) in 2012 (*p* < 0.0001). The major mutations Y181C and K103N were most prevalent, and were present in 27 and 20% of all specimens respectively. Two thirds of specimens with NNRTI resistance had a single mutation detected. NRTI resistance was present in 10 (5%) specimens, 4 each in 2010 and 2011, and 2 in 2012. Of these, the M184V/I mutation was present in 6 sequences. All 10 specimens with detectable NRTI resistance mutations contained NNRTI resistance i.e. were resistant to both the NRTI and NNRTI drug classes. PI mutations were uncommon: the PI mutation M46I was detected in 2 specimens, and ≤ 2 accessory PI mutations were detected in 12 sequences (L10IV, V11I, K20 M and A71V). All specimens clustered with HIV-1 subtype C apart from 1 specimen which clustered with HIV-1 subtype D.

Non-amplification of 47 specimens was not associated with infant ART exposure status (*p* = 0.268) but was associated with year of specimen collection (*p* < 0.001, data not shown). Genotyping PCR amplification in 2012 was 31% lower than previous years, but was consistent across all exposure categories. Rates of NNRTI resistance detection were not affected by changes in any ARV exposure categories over the years (*p* = 0.722, data not shown).

Analysis of infant resistance in relation to antiretroviral drug exposure demonstrated that NNRTI resistance was detected in 74% (95% CI [56–87%]) of infants exposed to maternal ART plus infant ARV (daily NVP ± AZT), and in 68% (95% CI [55–79%]) of infants exposed to maternal ARV prophylaxis plus infant ARV, Table 2. In addition, 64% of infants who were not exposed to maternal ART/ARV but received daily infant NVP ± AZT harboured NNRTI resistance. NNRTI resistance was also present in 26% of infants with no or unknown exposure to any ARVs. NRTI resistance was more prevalent in infants exposed to maternal ART (15%, *p* = 0.034), compared with other PMTCT regimens, Table 2.

## Discussion

We analysed infant HIV drug resistance using specimens from the 2010, 2011–12 and 2012–13 nationally representative South African PMTCT surveys. These measured 3.5, 2.7 and 2.6% MTCT at 4–8 weeks postpartum respectively [5, 7, 8]. Mutations associated with HIVDR were detected in half to two-thirds of the HIV-1 infected infants participating in these surveys. Prevalence of resistance was lower in 2010 (37%) compared to 64 and 63% in 2011 and 2012, respectively. This corresponded to a period of transition from a national PMTCT policy that recommended ART for women with CD4 cell counts ≤200 cells/mm^3^ or ARV prophylaxis from 28 weeks with single dose NVP to one that recommended maternal ART from as soon as possible after diagnosis for women with higher CD4 cell counts (≤350 cells/mm^3^) with six weeks infant NVP or maternal ARV (AZT) prophylaxis from 14 weeks, and daily infant NVP throughout breastfeeding (Option A). However, prevalence of resistance increased to 64 and 63% in 2011 and 2012 when the national policy had fully transitioned to PMTCT Option A, with increased infant NNRTI post-exposure prophylaxis (six weeks or more compared with sdNVP in the previous policy). Resistance to the NNRTI drug class only, which was part of all PMTCT policies in place during these surveys, with increasing infant NNRTI exposure between 2010 and 2012, was most commonly detected, whilst dual NRTI-NNRTI resistance was low (~ 5%) of specimens overall. The major NNRTI mutations Y181C/I and K103N were the most commonly detected mutations. Resistance to PIs was negligible. Paediatric antiretroviral treatment policies recommend abacavir (NNRTI), 3TC (NNRTI) and Kaletra (PI) in children < 3 years, or abacavir (NNRTI), 3TC (NNRTI) and efavirenz (NNRT) in children > 3 years, therefore we postulate that the increased NNRTI resistance would not impact the efficacy of the PI-containing treatment regimens used to treat early (< 3 years of age) paediatric management within the country. Whilst the NEVEREST study demonstrated that NNRTI resistance could re-emerge after a wash-out period, following re-exposure to the selective pressure of the NNRTI, we do not know what impact this has at programmatic level, in the current setting where children between 42 weeks gestation and 3 months of age are often treated with NVP-containing regimens, before switching to PI- (lopinavir/ritonavir)-containing regimens. None of the infants in our setting were on treatment at the time of HIV diagnosis.

Detection of resistance in infants was more common amongst those with documented exposure to maternal and/or infant PMTCT prophylaxis, in that more than two thirds of infants exposed to PMTCT harboured resistance. However, among infants with no documented exposure to PMTCT interventions resistance was detected in 26% of infants.

These findings are similar to those reported from other studies performed in the southern African region over the same time frame. NNRTI resistance was detected in 57% PMTCT-exposed HIV-infected infants aged ≤2 years (median age 19 weeks) in the Johannesburg area in 2011 [18]. National surveys to assess resistance levels in infants ≤18 months of age in Swaziland (2011, median age 4 months) and Zimbabwe (2010, median age 4 months) showed NNRTI HIVDR in 32 and 63% of participants, respectively [19]. In these studies, NNRTI resistance was more prevalent in infants exposed to maternal ART and / or extended infant NVP, and decreased with age. The data contained within this study are unique in that proportions of resistance in younger infants predominantly at 6 weeks of age are reported, drawn from a nationally representative sample of HIV PCR positive infants, who would have been recently exposed to PMTCT interventions.

Drug resistance was further detected in a quarter of specimens with no or undocumented exposure to PMTCT interventions, again similar to previous reports, wherein HIVDR was detected in 9–50% of infants with undocumented PMTCT exposure [18, 19], possibly due to maternal transmission of a resistant virus. These children are at higher risk of virological failure [3, 20, 21]. As levels of pre-treatment resistance in children increases [22], early implementation of fully active regimens in HIV-infected children remains a priority for treatment programs.

The low rate of specimen amplification in the 2012 specimen set relative to those collected during the other surveys is concerning, but there does not appear to be significant changes in infant exposure status over the years, nor did this lower rate appear to have had an effect on resistance prevalence estimates. However, the actual numbers tested for resistance in each exposure category by year were small, limiting modelling efforts to determine associations between years and exposure categories.

The study is limited by reliance on self-reporting of maternal and infant PMTCT prophylaxis during the time of data collection for the current pregnancy, and lack of information on possible PMTCT exposure during any prior pregnancies. The study further analyses data from infants predominantly exposed to dual prophylaxis or option A and therefore does not provide an understanding of the impact of prolonged daily NVP exposure or option B+ on the prevalence of HIV-1 drug resistance in infants beyond 8 weeks of age. In addition, conventional specimen sequencing strategies were employed to detect resistance mutations in the iDBS specimens. It can be expected that more sensitive technologies such as next generation sequencing will increase the frequency of drug resistant variant detection in these studies.

## Conclusion

Data from these national surveys illustrate the complex drug-resistance challenges that arise as more efficacious antiretroviral therapy is introduced and at earlier time points. With the policy change to PMTCT Option B+ (life-long ART for all HIV positive pregnant and lactating women), efforts to administer treatment early, achieve optimal adherence and monitor virological suppression regularly during pregnancy and lactation are needed to facilitate the elimination of MTCT. In the event of vertical HIV transmission such efforts may prevent maternally-acquired drug resistance or the development of infant HIV drug resistance following ARV exposure.

Whilst PMTCT programs have achieved significant reductions in levels of infant HIV infection, these programs should strive to provide optimal care, adherence counselling and virological suppression to pregnant women to further reduce the risk of HIV transmission to infants, and especially the transmission of resistant virus. The high rate of resistance in infants with no or unknown exposure further confirms that history of PMTCT is not the only predictor of resistance. Additionally, the low frequencies of PI resistance, supports the recommendation to treat all infants infected with HIV with PI-based regimens at the outset; however, the poor palatability of PI’s, and questionable safety of PIs in children under the age of 2-weeks to three months and in sick and premature infants, preclude their unconditional use. In the context of PMTCT Option B+, more data are needed on the relationship between maternal ART duration, maternal ART adherence and the transmission of resistant virus to the infant, versus transformation of virus into a resistant form due to infant drug exposure.

## Data Availability

The sequences analysed in this manuscripts are available in NCBI Nucleotide Database GenBank under accession numbers MH568836 - MH569053.

## Abbreviations

3TC: Lamivudine
ART: Antiretroviral therapy
AZT: Azidothymidine
d4T: Stavudine
EFV: Efavirenz
FTC: Emtricitabine
HIV: Human immunodeficiency virus
HIVDR: HIV drug resistance
iDBS: Infant dried blood spot
MTCT: Mother-to-child transmission of HIV
NICD: National Institute of Communicable Diseases
NNRTI: Non-nucleoside reverse transcriptase inhibitor
NRTI: Nucleoside reverse transcriptase inhibitor
NVP: Nevirapine
PCR: Polymerase chain reaction
PI: Protease inhibitor
PMTCT: Prevention of mother-to-child transmission of HIV
SAPMTCTE: South African prevention of mother-to-child transmission of HIV programme evaluation
Sd-NVP: Single dose Nevirapine
TDF: Tenofovir
TNA: Total nucleic acid
